# Left Upper Lobectomy for Congenital Lobar Emphysema in a Low Weight Infant

**DOI:** 10.1155/2016/4182741

**Published:** 2016-08-15

**Authors:** Meletios Kanakis, Konstantinos Petsios, Dimitrios Bobos, Kosmas Sarafidis, Stefanos Nikopoulos, Konstantinos Kyriakoulis, Achilleas Lioulias, Nicholas Giannopoulos

**Affiliations:** ^1^Department of Pediatric and Congenital Heart Surgery, Onassis Cardiac Surgery Center, Athens, 17674 Kallithea, Greece; ^2^Department of Neonatology and Neonatal Intensive Care Unit, Aristotle University of Thessaloniki, Hippokration Hospital, 54642 Thessaloniki, Greece; ^3^Department of Thoracic Surgery, Sismanoglio General Hospital of Athens, 15126 Marousi, Greece

## Abstract

Congenital lobar emphysema (CLE) is a rare lung congenital malformation. Differential diagnosis of the disease remains challenging in an infant with acute respiratory distress. We report a case of a 3-week-old female infant with a weight of 2.1 kg who presented respiratory distress related to CLE. Left upper lobectomy was performed and she had an uneventful recovery.

## 1. Introduction

Congenital lobar emphysema (CLE) is a rare anomaly of lung development and often appears in the neonatal period, with hyperinflation of one or more pulmonary lobes, in the absence of bronchial obstruction [1]. Actually, true emphysematous changes are lacking and some investigators classify this disease as congenital hyperinflation. CLE occurs in 1 case per 20–30 thousand births [2]. The etiology of congenital lobar emphysema is idiopathic in half of cases, whereas the other 50% have several mechanisms proposed to explain the air-trapping, which can be divided into intrinsic and extrinsic subtypes [3].

Early diagnosis is crucial and in many cases it is difficult to differentiate between CLE and hyperinflation resulting from extrinsic bronchial obstruction (lymph nodes, vessels, masses, or cysts) that compresses the bronchus and produces valve obstruction. However, it is stated that even more than half of CLE cases are not related to airway abnormalities. Surgical removal is the most common treatment choice with operative mortality rate about 3 to 7% [2].

## 2. Case Presentation

**Figure 1:**
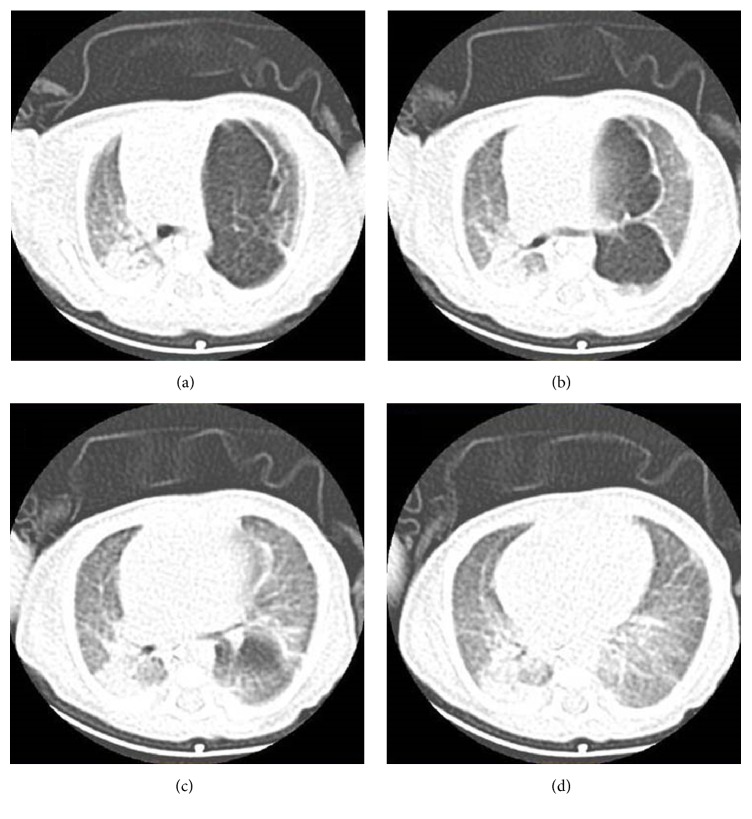
Computed tomography of the chest showing the presence of an emphysematous left upper lobe and atelectasis of the left lower lobe with subsequent contralateral mediastinal shift to the right causing atelectasis of the right lung.

A 3-week-old female infant with a weight of 2.1 kg was referred to our Department for the surgical management of CLE. The infant was in respiratory distress. Oxygen saturation was 82% in room air. Past medical history was unremarkable. In the last week, mother had noticed occasional episodes of tachypnea. Rapid development of dyspnea episodes brought the infant to the Emergency Department in respiratory distress. Respiratory rate was 70/min and oxygen saturation was of 85% on room air. Chest examination showed asymmetry with bulging of left hemithorax with decreased air entry on the left and crepitations on the right. Chest X-ray showed overdistention of the left lung field with asymmetry in parenchymal transparency with a hyperlucent zone. A prominent mediastinal shift with compression and atelectasis of the right lung was evident. Initial diagnosis was consistent with respiratory tract infection complicated with pneumothorax. Further diagnostic workup with computed tomography scan confirmed the presence of an emphysematous left upper lobe and atelectasis of the left lower lobe with subsequent contralateral mediastinal shift to the right causing atelectasis of the right lung (Figures 1(a) and 1(b)). Diagnosis was consistent with CLE. Echocardiographic workup showed the presence of small patent ductus arteriosus (PDA), which was hemodynamically insignificant.

**Figure 2:**
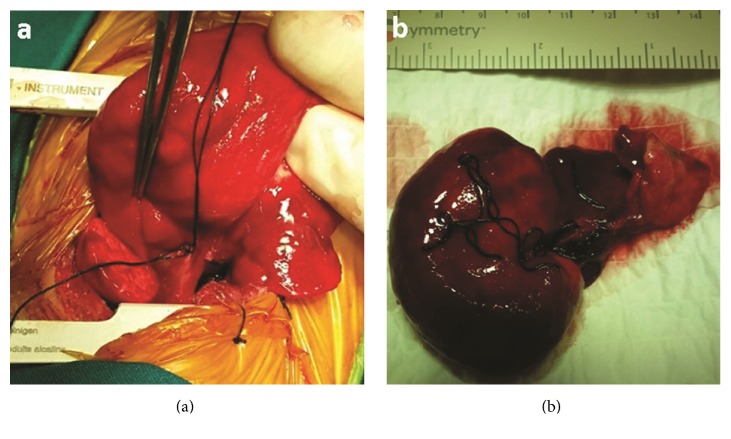
Intraoperative photos showing the macroscopic appearance of the hyperexpanded left upper lobe.

Decision was favorable for surgical treatment due to the presence of respiratory distress in the setting of mediastinal shift and subsequent compression of the unaffected lung lobes. She underwent a left posterolateral thoracotomy and a left upper lobectomy was also performed (Figures 2(a) and 2(b)). Patent ductus arteriosus was also ligated. She had an uneventful postoperative course and she was discharged from the hospital on postoperative day 12. Pathology examination of the resected lobe revealed lung parenchyma with atelectatic changes and emphysematous dilatation of alveolar spaces.

## 3. Discussion

Nowadays, CLE is usually being diagnosed during prenatal evaluation by ultrasonography and may be associated with polyhydramnios and fetal hydrops. Early diagnosis of CLE is crucial and in many cases is complicated due to the variety of its clinical presentation that varies from mild tachypnea to severe respiratory distress [4]. The most common clinical presentation is neonatal acute respiratory distress, which is caused by localized air trapping that compresses the ipsilateral and contralateral normal lungs. Symptoms worsen as the emphysematous lobe gradually enlarges. Cyanosis is the second most common finding. However, similar symptoms with CLE may occur in bronchopneumonia, cyanotic congenital heart diseases, and several congenital abnormalities of the lung. Congenital lobar emphysema may be confused with tension pneumothorax. Chest tube insertion may further increase respiratory distress and lead to injury of the lung parenchyma. In CLE the pulmonary vessels extend to the periphery of the hyperinflated lobe and there is no visualization of a pleural line unlike in pneumothorax [5]. Moreover, differential diagnosis includes congenital cystic adenomatoid malformation, sequestration, bronchogenic cyst, unilateral hyperlucent lung syndrome, and pulmonary interstitial emphysema.

The diagnosis, although not definitive, is made by clinical examination and chest X-ray and can be confirmed by CT, as we performed in our case. Chest radiograph usually shows many abnormalities that raise the suspicion of CLE. In addition, CT provides anatomic details that serve as a guide for safe resection of the affected lobe. CT can show the abnormally narrowed bronchus and affected lobe, as well as the collapsed lobe. It is also useful in differential diagnosis of mediastinal mass or an enlarged heart and may rule out the presence of associated anomalous vascular anomalies [6].

In literature the use of bronchoscopy as an important tool in the differential diagnosis is also discussed, but it is not recommended for primary screening test and it is mainly indicated for children whose symptoms appear in later neonatal period [2]. Furthermore, improper use of this procedure may aggravate the respiratory distress in CLE patients. On the contrary, Tey et al. in a recent article concluded that flexible bronchoscopy should be used to study suspected CLE cases to determine their causes and decide whether to treat the patient conservatively, by lobectomy, or by other strategies [7].

In our case the clinical presentation of CLE occurred during the third week of life with extreme respiratory distress. As with this child, respiratory distress is the commonest mode of clinical presentation. Dyspnoea, wheezing, grunting respiration, tachypnea, and sometimes progressive cyanosis are the most common symptoms. In almost 95% of CLE cases the clinical signs are evident in the early neonatal period (from few days after birth to six months) but there are a number of cases for which diagnosis may be delayed up to 5-6 months [5, 8].

The low weight of the child was a challenge for both anesthesia and surgical procedure. The various factors that increase morbidity and mortality in these patients are usually consequent to the immaturity of the various systems and the associated congenital defects. Infants with low body weight undergoing thoracic surgery are a major challenge for both surgeons and the postsurgery care unit. It has been observed that postsurgery outcome for low weight infants is worse compared to the outcome of normal body weight infants. Therefore, the need for close monitoring perioperatively and postoperatively is greater whereas procedure complications may occur. CLE perioperative mortality ranges from 3 to 7% [9]. Due to the low weight of the child we focused not only on the appropriate respiratory weaning but also on the prompt nutrition intake.

The anesthetic considerations in neonatal surgical emergencies are based on the physiological immaturity of various body systems, poor tolerance of the anesthetic drugs, associated congenital disorders, and concerns regarding the use of high concentration of oxygen. Regarding the induction of anesthesia when positive pressure ventilation is applied before opening of the chest, it may cause rapid inflation of emphysematous lobar cyst with sudden mediastinal shift and cardiac arrest. Therefore, induction of anesthesia should provide adequate spontaneous ventilation with minimal airway pressure. Occasional gentle assistance is necessary. Once the chest is opened and the affected lobe is delivered, the patient can be paralyzed and the lungs can be ventilated by controlled ventilation [10]. In our case, manual-assisted ventilation with low inflating pressure (7–18 cm H_2_O) was used until the chest was opened.

Congenital heart disease has an association with congenital lobar emphysema. In literature 12–20% concomitant CHD or vascular slings have been reported. Therefore, many studies recommend the performance of echocardiography during the differential examination [1, 3, 4]. In our case echo revealed only the presence of a PDA. Although it was not hemodynamically significant, PDA ligation was performed after the completion of lobectomy via the left posterolateral thoracotomy.

The lobar emphysema was located in the left upper lobe and this is the most frequently affected site. According to previous studies, left upper lobe is most often involved, followed by the right middle lobe [1, 4, 11]. The involvement of the lower lobes is very rare. The cause of distribution of the affected lobes is not well known. It is possible that location is related to embryonic stage [7]. Moreover, in many cases a shift of the mediastinum occurs as was the case here.

Surgery is the treatment of choice, especially in the setting of mediastinal shift with subsequent compression of the unaffected lung lobes. In earlier case series surgical excision of the affected lobe was recommended as soon as possible in all infants younger than 2 months and in infants older than 2 months who present severe respiratory symptoms. The clinical presentation of the infant was used as a guide to the appropriate time for surgery. In some cases with older infants, with mild or moderate respiratory distress, conservative management can be performed along with a close follow-up of the patient [2, 6]. In our case, although lobectomy in an infant with a low weight was more demanding, postoperative course was uneventful with an excellent outcome.

The diagnosis of CLE in infants may be a challenge. The choice of treatment should be based on the severity of onset. This case suggests that lobectomy could be safely performed in a low weight infant with severe respiratory distress.
